# Barriers and enablers to delivery of the Healthy Kids Check: an analysis informed by the Theoretical Domains Framework and COM-B model

**DOI:** 10.1186/1748-5908-9-60

**Published:** 2014-05-23

**Authors:** Karyn E Alexander, Bianca Brijnath, Danielle Mazza

**Affiliations:** 1 Department of General Practice, School of Primary Health Care, Monash University, Building 3, 270 Ferntree Gully Road, Notting Hill, Melbourne, Victoria 3168, Australia; 2 NHMRC Early Career Public Health Fellow, Department of General Practice, School of Primary Health Care, Monash University, Building 1, 270 Ferntree Gully Rd, Notting Hill, Melbourne, Victoria 3168, Australia; 3 Department of General Practice, School of Primary Health Care, Monash University, Building 1, 270 Ferntree Gully Rd, Notting Hill, Melbourne, Victoria 3168, Australia

## Abstract

**Background:**

More than a fifth of Australian children arrive at school developmentally vulnerable. To counteract this, the Healthy Kids Check (HKC), a one-off health assessment aimed at preschool children, was introduced in 2008 into Australian general practice. Delivery of services has, however, remained low. The Theoretical Domains Framework, which provides a method to understand behaviours theoretically, can be condensed into three core components: capability, opportunity and motivation, and the COM-B model. Utilising this system, this study aimed to determine the barriers and enablers to delivery of the HKC, to inform the design of an intervention to promote provision of HKC services in Australian general practice.

**Methods:**

Data from 6 focus group discussions with 40 practitioners from general practices in socio-culturally diverse areas of Melbourne, Victoria, were analysed using thematic analysis.

**Results:**

Many practitioners expressed uncertainty regarding their capabilities and the practicalities of delivering HKCs, but in some cases HKCs had acted as a catalyst for professional development. Key connections between immunisation services and delivery of HKCs prompted practices to have systems of recall and reminder in place. Standardisation of methods for developmental assessment and streamlined referral pathways affected practitioners’ confidence and motivation to perform HKCs.

**Conclusion:**

Application of a systematic framework effectively demonstrated how a number of behaviours could be targeted to increase delivery of HKCs. Interventions need to target practice systems, the support of office staff and referral options, as well as practitioners’ training. Many behavioural changes could be applied through a single intervention programme delivered by the primary healthcare organisations charged with local healthcare needs (Medicare Locals) providing vital links between general practice, community and the health of young children.

## Background

Since 2007, significant reforms in Australia’s health and hospital system have shifted their focus towards prevention and a multi-sector government response, in a bid to improve healthcare and curtail the costs associated with an ageing population [1]. Local primary care organisations, known as Medicare Locals, are charged with providing the infrastructure to support identification of risk and implementation of preventive health programmes [2]. Outside the health system, educational reforms target early childhood and address intergenerational disadvantage [3]. These initiatives seek to improve health in early childhood, for despite Australia having one of the highest life expectancies world-wide, under-5 morbidity and mortality remains disproportionately high; 37% of Australian children suffer chronic health conditions and around 7% have a disability [4]. Additionally, 42% of 5-year-olds suffer dental caries [5], and more than a fifth of Australian children arrive at school developmentally vulnerable [6].

Although a review of the evidence for child health surveillance^a^ has found little evidence for effectiveness (principally due to a lack of clinical guidelines), the report concluded that there was a need to rethink how child surveillance was conducted [7]. Australia has a system of publically funded child health surveillance visits provided by Maternal and Child Health Nurses (MCHN) through local government. Delivery of services varies considerably state-wide, but in the state of Victoria – where this study was conducted – services engage more than 90% of families in a child’s first year. However, contact diminishes as the child gets older, so that by 3 ½ years of age, less than 60% of children complete health surveillance visits [8]. In contrast, general practice services are delivered from predominantly privately owned clinics. Rebates for services – inclusive of some preventive health assessments – are available from the national insurer ‘Medicare’ with the intent to secure universal access to subsidised primary care services. Consequently, more than 80% of the Australian population visit a general practitioner (GP) each year [9].

To increase opportunities for preventive health with young children, in 2008 ‘The Healthy Kids Check’ (HKC) [10], a one-off health assessment aimed at preschool children, was introduced into general practice, where 12% of GP-patient contacts are with children [11]. Administered by GPs and general practice nurses (PNs), the HKC comprises an assessment of growth and development, and offers health promotion opportunities (Table 1) on the occasion of a child’s preschool immunisations. Despite a Medicare rebate being applicable, uptake has been lower than anticipated, with only 16% of 4-year-olds completing a HKC in the first year. The state of Victoria ranked sixth out of seven states in terms of proportions of children receiving HKC services in 2012 [12]. Since its introduction, there have been no empirical studies examining the factors influencing uptake of the HKC in general practice.

**Table 1:** Components of the healthy kids check (2008)

**Mandatory**	**Non-mandatory**
Height	Discuss eating habits
Weight	Discuss physical activity
Eyesight	Speech and language development
Hearing	Fine motor skills
Oral health	Gross motor skills
Question toilet habits	Behaviour and mood
Note allergies	Other examinations as necessary

Barriers to the consistent delivery of preventive care for young children, prior to the introduction of the HKC, included insufficient time, poor financial reward, and a lack of community resources (*e.g*., information and referral services) [13]. Studies from the United States (US) have identified practitioner barriers to the US system of ‘well-child care.’ These include knowledge gaps, lack of confidence using validated tools [14,15], insufficient understanding of early intervention [16] (which hinders detection of developmental delays), inadequate office staff and poor remuneration [12]. For parents in Australia, our previous research showed that parent decision-making around accessing preventive care for their children was influenced by the birth order of the child, cultural health beliefs, healthcare costs, and limited knowledge about early intervention [17].

Therefore, for an increase in HKC services to occur, the behaviour change processes of several interacting groups of people, including parents and healthcare providers, operating at various organisational levels, needs to be considered. The development of such a ‘complex intervention’ must be underpinned by local evidence and rigorous psychological theoretical constructs, to both facilitate behaviour change and provide an explanation for the mechanism of change. The use of a theoretical framework in the design and evaluation of interventions has been increasingly emphasised by implementation researchers [18-20]. Guidance from the United Kingdom’s Medical Research Council proposes that where psychological theory underpins the iterative processes involved in designing a complex intervention, innovation is more likely to succeed [21].

**Figure 1:**
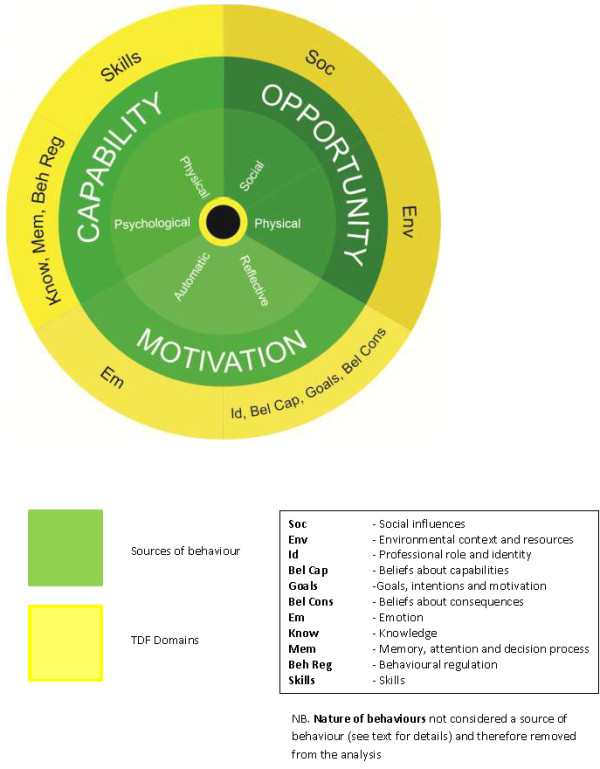
**Map of Theoretical Domains Framework (TDF) to Sources of Behavior on COM-B System **[26]**.**

The Theoretical Domains Framework (TDF) is a method established to understand behaviours theoretically so that processes can be effectively targeted for change [19]. The original TDF^b^ consisted of 12 domains and was developed by consensus from a combination of 33 psychological and organisational theories to provide a guide towards implementing evidence-based practice (Table 2) [19]. This approach seeks to make psychological theory more accessible to health service researchers. The TDF has been widely implemented across a variety of settings [22] and includes analysis of preventive health including preconception care [23], hand hygiene behaviours in a hospital setting [24], and human papilloma virus counselling in primary care [25]. The 12 domains of the TDF can be condensed into three core components: capability, opportunity and motivation (Figure 1) [26]. The COM-B model demonstrates that human behaviour (B) results from the interaction between personal physical and psychological capabilities (C), to utilise social and environmental opportunities (O) via motivators (M) that are reflective (thinking with the head) or automatic (emotional-‘thinking’ with the heart).

**Table 2:** **The theoretical domains framework (Michie 2005)**[19]

**DOMAINS**	
Knowledge	Memory, Attention and Decision processes
Skills	Environmental Context and Resources
Social/professional role and identity	Social Influences
Beliefs about capabilities	Emotion
Beliefs about consequences	Behavioural Regulation
Motivation and goals	Nature of the Behaviours

Utilising the TDF and COM-B, the aims of the present study were to determine the barriers and enablers to delivery of the HKC, and to inform the design of an intervention to promote provision of HKC services, in Australian general practice.

## Method

### Design

Focus group discussions with GPs and PNs were undertaken to explore their knowledge-behaviour gaps. Group dynamics were viewed as more likely than individual interviews to reveal attitudes and experiences, and the underlying reasons for specific behaviours [27].

### Sample

Three groups of GPs and three groups of PNs (total 40 practitioners) were recruited from three socio-culturally diverse urban areas of Melbourne, Victoria, broadly categorised as high income (Bayside), low income (Westgate), and culturally diverse (Dandenong). The study was advertised by newsletter and invitations were faxed to clinics served by Medicare Locals in these areas. To increase responses, phone calls were made to individual practice managers and PNs by one of the researchers (KA), and participants could recommend other practitioners (snowballing), with a limit of one GP and one PN from each clinic.

### Procedure

An interview guide based on the TDF was designed to prompt focus group discussions (Table 3). To avoid ‘group hierarchies’, focus groups were divided by practitioner (except for one attendee, a practice nurse, who opted to attend the GP group) (Table 4). Focus groups took place between June 2011 and October 2011 (three years following introduction of the HKC), lasted approximately 90 minutes and were facilitated by the first two authors (who declared their positions), one a GP, trained in qualitative research methods, the other, an experienced qualitative researcher. A voucher valued at $200 for GPs and $80 for PNs was given to each practitioner in appreciation of their time. This incentive discrepancy reflects differences in average earnings between practitioner groups and known difficulties with recruiting practitioners to research projects [28]. Focus groups were audio-recorded and later transcribed for analysis. A report was emailed to each participant to solicit feedback. Ethics approval was obtained from Monash University, and all participants provided written informed consent.

**Table 3:** Prompts for focus groups according to Michie’s theoretical domains

**Theoretical domains**	**Examples of interview prompts**
Knowledge	Do you know about the mandatory and non-mandatory components of HKCs? Do you know about the RACGP guidelines for child preventive health?
Skills	How have you learned how to do a HKC? Have you had any training for HKCs?
	Which components of the HKC do you perform? Are there any specific areas of difficulty?
	One of the non-mandatory components is questioning the social and emotional behaviour. Do you ask about that?
	Can you assess the social and emotional well-being of a three-year-old?
	What do you think about measuring children and calculating BMI?
Social/professional role	Who do you think should be doing HKCs?
	How do they fit with the checks done by MCHNs?
	Do you think general practitioners have a role in preventive health in general?
	Why did you set up HKCs in your practice?
Beliefs about capabilities	How good are we at picking up problems in young children?
	How easy or difficult is it to do a HKC?
	Do you think that you’ve got the skills (to do a HKC)?
	Do you fear that you might miss something? How confident are you that you can pick up a problem?
	How confident are you with the assessment of social and emotional wellbeing
Beliefs about consequences	Do you think HKCs are worthwhile? Do you think they should be scrapped?
	In your experience of doing health checks with this age group, did you come across problems in your population?
	What do you think about the evidence base behind the HKC?
	How do you think parents view the HKC? Has anyone refused a check?
Motivation and goals	Why do you do HKCs? Why don’t you do HKCs?
Memory, attention and decision processes	Is performing a HKC something you usually do?
	Do you use any prompts?
	Has anyone decided NOT to do a HKC?
Environmental context and resources	Do you have any systems in place to run a HKC?
	Do you have the equipment? What do you use to help with a HKC?
	Is anyone using any questionnaires or tools with a Healthy Kids Check?
	Is there anything specific about WHERE you practice-your population group?
Social influences	Has anyone used any reminders or invitations for HKCs or do you just wait for people to ask?
	What do you think about the policy change that links the HKC with the Family Tax Benefits?
Emotion	How do you feel about health assessments with children? Does it give you any particular feelings or emotions?
Behavioural regulation	Are there procedures or ways of working that encourage you to do HKCs?
Nature of the behaviours	What do you currently do about HKCs
	What about weighing an overweight child? How do you approach an overweight child?

HKC: Healthy Kids Check; RACGP: Royal Australian College of GPs; MCHN: Maternal and Child Health Nurse.

**Table 4:** Focus groups according to practitioner and area

**Name and description of area of Melbourne**	**Participant numbers in GP focus groups (Total =22)**	**Participant numbers in practice nurse focus groups (Total =18) (all female)**
Bayside upper socio-economic	6 (3 female 3 male)	6
Dandenong lower socio-economic Culturally and linguistically diverse	9 + 1 practice nurse (6 female 4 male)	6
Westgate lower socio-economic	7 (4 female 3 male)	5

### Analysis

Data were analysed by applying categories from the TDF in a recursive process that followed the customary steps of thematic analysis [29]. Specifically, after reading through the entire dataset, the first two authors independently coded the data from each transcript and assigned initial ‘code names,’ then collaborated and discussed choices, with a third researcher available to resolve any differences in opinion. Data were imported into NVivo 8 [30] and de-identified. After agreement had been reached, an additional step was taken to match code names to themes represented by the ‘domains’ within the TDF. This required the researchers to re-read data within the codes, then allocate the codes to the appropriate domains. This sometimes meant that the data coded under one code name was categorised into two or three different domains within the TDF. All codes could be applied to at least one domain. From there the domains were mapped to the COM-B system (Table 5).

**Table 5:** Mapping of codes to themes from Theoretical Domains Framework (TDF) and COM-B system

**Code assigned directly to transcripts from focus groups**	**Themes from TDF**	**COM-B system**
Rationale for doing HKCs**	Knowledge	Psychological CAPABILITY
Memory-remembering to do HKCs/preventive	Memory, attention and decision processes	
Growth and weight component of HKC**		
Systems and prompts**	Behavioural regulation	
Structure-logistics (how the clinic is run)***		
Tax incentive issues prompting HKC		
Standardisation of HKCs or components within		
Medicare and item numbers		
Immunisation or vaccination issues		
Financial barriers (for practitioners)		
Dental component of HKC**	Skills	Physical CAPABILITY
Eye or vision component of HKC**		
Hearing component of HKC**		
Child support network, *e.g*., childcare & kinder**	Social influences	Social OPPORTUNITY
Parent concern		
Role of MCHN		
Population screening		
Socio-cultural issues		
Resource allocation as equity/ethical concern**		
Systems and prompts for HKCs**	Environmental context and resources	Physical OPPORTUNITY
Structure-opportunistic (appointments)**		
Structure-logistics (how the clinic is run)***		
Structure- IT		
Space and resources including ‘Purple Book’		
Time barrier		
Dental component of HKC**	Beliefs about capabilities	MOTIVATION- Reflective
Eye or vision component of HKC**		
Social & emotional health component of HKC***		
GP knowledge and skills**		
PN attitude and feelings**		
PN knowledge and skills		
Role of the PN**		
PN attitude and feelings**	Professional role and identity	
Role of the PN**		
GP attitude and feelings		
Role of GP		
Social & emotional health component of HKC***		
Child support network, *e.g*., childcare & kinder**		
Motivation (to do HKC or preventive care)	Motivation and goals	
Preventive healthcare		
Rationale for doing HKCs**	Beliefs about consequences	
Outcomes from HKCs		
Early intervention		
Bureaucracy and ‘red tape’ barriers		
Social & emotional health component of HKC***		
Growth and weight component of HKC**		
Resource allocation as equity/ethical concern**		MOTIVATION-Automatic
Hearing component of HKC**	Nature of behaviours	Not included in COM-B model but each code is a duplicate
GP knowledge and skills**		
Structure-logistics (how the clinic is run)***		
Structure-opportunistic (appointments)**		

**mapped to two different themes from TDF; ***mapped to three different themes from TDF.

There were two domains of the TDF that we did not match any data to: the ‘Emotional/Automatic’ aspects of ‘Motivation’ and the ‘Nature of behaviours’. With regard to the former domain, although specific questions had been asked about emotions felt by practitioners when dealing with young children and health screening, responses were captured under the theme ‘GP attitudes and feelings’. This was assigned to the domain, ‘Professional role and identity’, and ultimately mapped to ‘Motivation’ in the COM-B model. The domain ‘Nature of behaviours’, part of the original list of domains within the Framework, could not be assigned to the COM-B model because whilst it described context (current practice), it did not provide a source of behaviour. This domain was subsequently removed in a review of the Framework which tested its validity with a second group of behavioural change experts [31].

## Results

Focus group captured a diverse range of practitioner experiences with the HKC, in each study area: some had not, as yet, provided a single HKC, others delivered a few checks occasionally, and some practices regularly booked HKC appointments or extended to provide entire clinics of HKC services. The study found that, overall, practitioners reacted positively towards providing preventive healthcare to young children. They conceptualised this in terms of the provision of immunisation services and HKCs and, to a lesser extent, opportunistic growth and developmental assessments during ‘sick-child’ consultations. Below we describe how our data aligns within the TDF and COM-B model (Table 6).

**Table 6:** Summary of the evidence, application of TDF and COM-B and proposed interventions

**Evidence**	**TDF**	**COM-B**	**Proposed intervention**
	**Capability**		
GPs did not always know how to assess aspects of development	Knowledge	Capability-Psychological	Education and training which incorporates:
PNs did not know how to do HKCs (until they had received training)			Knowledge about “Early Intervention”
GPs did not always *remember* how to assess overall development	Memory		Physical examination techniques
GPs conducting HKCs were uncertain about which tests to use and how to do them	Physical skills	Capability-Physical	Structured developmental assessment and evidence behind this
			Interpersonal skills training
PNs wanted training on skills required for HKCs			Tools appropriate to primary care
PNs did not know how to manage parent reactions to possibility of abnormality in child’s development.	Interpersonal skills	Capability-Psychological	
Variable quality of HKCs	Behavioural regulation		
	**Opportunity**		
Equipment barriers	Environmental context and resources	Physical opportunity	Funding for equipment and tools, including information technology
Supportive health promotion brochures			
Space in clinic to accommodate the HKC examinations			Provision of health promotion literature
Medical contact with children especially vaccinations	Social influences	Social opportunity	Education and training which incorporates:
			Practice structure
Employing a PN			Office systems including recall and reminder
Having staff responsible for managing a recall system			Tools appropriate for use in general practice (time saving)
Having a “HKC Champion”			
The professional mix in the practice			
Competing interests of practice population healthcare needs			
Practitioners had insufficient time			
“Healthy Start for School”-Tax incentive to complete HKC			Strengthen government support for delivery of early childhood intervention across services
Increase in Medicare rebate			
Belief that general practice competes with other service providers to provide HKCs			
	**Motivation**		
Belief that MCHNs have ownership and expertise in preventive healthcare for young children	Professional role and identity	Reflective motivation	Education and training which address capability and professional roles with task delegation
GPs find process tedious and place HKCs low priority			
Alternative model of developmental assessment with early childhood educators playing primary role			
Developing the role of the PN in Australian general practice	Professional role and identity & Beliefs about capabilities		
PNs expressed low levels of self-confidence with some of the components of the HKC			
PNs preferred clear boundaries when delivering HKCs			
PN personal drive for professional development	Goals, intentions and motivation & Positive beliefs about consequences		Opportunity to build capacity in early childhood development involving other professionals
HKCs used by some practitioners to develop professional expertise			
PNs more confident about their abilities were more satisfied with outcomes			Centralisation and dissemination of information about community resources
Outcomes and referral pathways are important to practitioners beliefs			
GPs expressed low confidence with evidence behind HKCs	Negative beliefs about consequences		
Belief that timing of HKC is too late for early intervention			

### Capability

#### Skills, knowledge, memory, attention and decision processes

Practitioners’ self-assessment of their capabilities to screen the health of young children varied, but all practitioners held reservations about particular components of the HKC. GPs generally thought that they had sufficient skills and knowledge, but when challenged to consider each component of the HKC, they were uncertain about how to test the vision, hearing and social-emotional health of this age group, and admitted to difficulties recalling child developmental stages. PNs were not prepared to conduct HKCs until they had received specific training and expressed concerns that they risked antagonising parents if they suggested a child’s development deviated from normal, particularly with behavioural problems, social and emotional difficulties, weight and body mass index:

I don’t think I’m equipped to assess a four-year-old enough, even though I have had two [children]. I don’t feel comfortable sometimes … talking to parents if there’s issues. It’s quite daunting… parents don’t like to hear that something’s wrong with their child. (PN1 Dandenong).

How do you, if you’ve got an overweight 3 ½-year-old, if you’ve not got the training? How do you deal with that without really offending the parent? (PN1 Bayside).

#### Behavioural regulation

Practitioners perceived that there could be a wide variability in the quality of HKCs and thought they should be standardised for greater consistency across practices and between practitioners. Participants compared the structure of primary healthcare in Australia, the United Kingdom and New Zealand; they believed the fact that individual practices were not held to account for the public health of a local population called for greater regulation of clinical behaviours.

It’s not like in England where you have a list and you know who’re your customers … We know patients float around here, there and everywhere, especially with kids … if you’re going to do it properly you do it in a programmed, reproducible, managed [way]. (GP1 Bayside).

Using specific screening tools was put forward as one solution to counter variability in practice and regulating standards.

Give us the tool …a tool that everybody can use. The same tool, because if we’re all doing it separately then where’s the base to start from? (PN2 Dandenong).

#### Opportunity

Practitioners who had the capabilities to conduct HKCs were either encouraged or deterred from performing HKCs according to conditions established by the physical and social environment, within and external to their clinics. These shaped the opportunities for establishing systems and conducting HKCs from their practices.

#### Environmental context and resources

Practitioners believed that computerised prompts worked to promote the delivery of HKCs but recalled many physical barriers including cost and difficulty accessing screening tools. For example, one PN had tried to source eye charts and was told by the company that the charts were only available to government employed MCHN services. Lack of supportive health literature, especially the ‘demise’ of the ‘Purple Book’, was also a source of much discussion. The purple-coloured booklet, entitled ‘Get Set 4 Life - Habits for Healthy Kids’, was initially allocated by the national government to support health promotion aspects of the HKC. However, by 2011, hard copies had run out and the book’s content moved online. Delivered in a child-friendly format containing cartoon characters and stickers, the book was viewed positively by practitioners because ‘It makes you feel like you’ve done something’ (GP1 Westgate).

PNs stated that the space to accommodate HKC examinations was inadequate at times. When an entire family attended for one child to have a HKC, conditions became cramped, and practitioners faced additional pressures as siblings quickly became bored and restless. PNs said such experiences undermined their professional image and left them feeling dissatisfied.

They’re often with other siblings and they’ve already been at the surgery half an hour, and by the end of it you’re feeling quite pressured for time and you can tell everyone is well and truly sick of this. (PN1 Westgate).

#### Social influences

Social structures within the practice influenced the delivery of HKCs, and two factors appeared essential: provision of vaccination services and employment of a PN at the practice. Where clinic protocols related vaccination services to the HKC, designated staff were often assigned to manage a system of invitations, recalls and reminders.

We send our recalls every couple of months, and we do have really good results from that. We have most people come back when they get their immunisations. (PN2 Westgate).

In these situations, PNs were ‘trained up’ to conduct HKCs, so that demand could be fulfilled:

They [GPs] want practice nurses to come in and drive all these things because they haven’t got the time. (PN2 Bayside).

Additionally, where a practice had a practitioner who ‘championed’ the promotion of preventive healthcare for young children, the clinic’s capacity to deliver HKCs increased. GPs who had a special interest in child health, for example, made particular efforts to accommodate HKCs or assessed child development opportunistically with vaccination consultations. The professional mix in the practice also influenced its provision. If a paediatrician or MCHN consulted from the same office space, practitioners believed this promoted the overall delivery of child preventive services, and they supported these shared care models.

In the broader social environment, recent fiscal policy changes were noted to influence parents’ uptake of HKCs. In 2011, the federal government determined that for a family to receive a particular tax-benefit, a health check had to be obtained for each child turning four years of age [32] (from a MCHN or GP). Practitioners generally agreed with this policy, and believed it encouraged the assessment of more vulnerable children.

Discussion about how increases in government rebates for HKCs (as they were brought into line with other health assessments and rebated according to time spent with the patient) had encouraged some practitioner’s efforts towards establishing services, also revealed perceptions of market competition:

(Laughing) The practice nurses in my practice at the beginning of this year were saying, ‘Oh, maternal and child health nurses, they’re in the best position to do it.’ We’re saying, ‘No, no we get money for this. We *need* to be doing this!’ (GP1 Dandenong)

The competing priorities of general practice: chronic disease management, health assessments for other population groups (*e.g*., aged-care assessments) and acute care needs, created time pressures, and highlighted social environmental barriers still remaining for practitioners.

#### Motivation

The evolution of respective practitioner roles around providing HKCs tied in with beliefs about capabilities and beliefs about the outcomes resulting from provision of preventive healthcare to young children. ‘Motivation’ is a key factor to understanding the uptake of HKCs.

#### General practitioners – professional role and identity

Many GPs struggled to understand why HKCs had been introduced, believing they acted as a ‘safety net’ to ‘catch’ those children who had missed out on MCHN services (Bayside GP discussion). They did not perceive themselves as being active participants in childhood surveillance, and found the HKC procedure to be tedious.

I didn’t do medicine to do four-year-old health checks … You could sit all day and do four-year-old checks, over-75 checks, over-45, you know… You want to see the acute illnesses. (GP2 Bayside)

Most GPs thought that the role of ‘screening’ young children belonged to MCHNs. Although they acknowledged they had a role to play in preventive health in general, one-off health assessments of preschool children were given a low priority, and their capacity was limited by competing and more urgent demands on their time.

If you give the GP’s so much prevention, because it’s the hugest, biggest, fattest end of the iceberg … it takes up so much time you don’t actually get to the other stuff and you have people dying at your door because they can’t get in. (GP1 Westgate)

Several practitioners proposed an alternative model of childhood surveillance that encompassed a secondary role for GPs, whereby identified problems could be referred to the GP for further assessment. They believed developmental problems would be more easily identified in group situations where children could be assessed against their peers. They targeted kindergarten teachers as being ideally placed to assess child development because they already had a role appraising children’s ‘school-readiness’.

#### Practice nurses – professional role and identity and beliefs about capabilities

PNs thought that their role in Australian general practice was still in its infancy (compared with places like the UK), and they talked about establishing a foothold in general practice and striving to project a professional image. They also believed that the provision of HKCs was the remit of the MCHN. The perception in one group discussion was that MCHNs ‘got their nose out of joint’ (PN3 Bayside) when HKCs were introduced, so that they had ‘retaliated’ with a radio advertising campaign. This inter-professional conflict created anxiety for PNs, and they conveyed low levels of self-confidence about their capacity to provide child health checks.

Given that the infant welfare centres do have the expertise, if I was a mother I know which one I’d rather go. (PN4 Bayside)

#### Goals, intentions and motivation and positive beliefs about consequences

Nevertheless, PNs also perceived that GPs wanted them to ‘drive’ the delivery of HKCs and, once training was offered and clear professional boundaries had been established, the opportunity to advance their professional standing motivated them towards providing services.

I remember saying at the beginning, ‘I don’t want to do them’ because I don’t know anything about them … and then they offered the education and I thought, ‘It’s a really good education, it adds to …my repertoire … my knowledge base.’ (PN3 Bayside)

Whilst the majority of practitioners were slow to embrace HKCs, a few readily used the provision of HKCs to support their personal professional development. Two GPs (one a GP in Bayside, the other in Westgate focus group), who had additional qualifications in paediatrics, sought children from vaccination consultations to opportunistically conduct developmental assessments or HKCs, and two PNs were independently conducting HKC ‘clinics’, without GPs, and had established clear referral pathways. These PNs were much more confident about their abilities and expressed more satisfaction that the problems they identified validated doing HKCs.

We have quite a few that go on for speech therapy or we have them on care plans because they’ve got learning difficulties or things like that … they’re able to access better services … Not everybody needs it … but the one or two that you do pick up that can get services, it makes it all worthwhile. (PN2 Dandenong)

Of interest was the fact that both of these PNs participated in the focus group in the Dandenong region, an area which serves a large migrant population of low socio-economic status.

#### Negative beliefs about consequences

Many practitioners, however, voiced concerns about the overall value of HKCs and low levels of evidence for childhood surveillance and screening.

[Chlamydia and bowel cancer screening] have an evidence-base to [them]… And then we have this healthy four-year-old test – but what’s the evidence base for this? (GP1 Dandenong focus group)

Practitioners recognised that ‘early intervention’ was important but felt defeated by the fact that the HKC was linked to immunisations given at four years of age, an age they considered too late for effective intervention before the start of school. Where services were difficult to access or where there was less certainty about what to do for ‘test-positive-children,’ practitioners were further disinclined to carry out HKCs. As one GP said, ‘So you find something wrong, but what’s the management after that?’ (GP2 Westgate). The ‘can of worms’ analogy captured their reticence and the opening up of a myriad of difficulties, particularly with social-emotional and behavioural health assessments. Some practitioners thought that particular parent groups would feel judged:

(GP2) Who will talk about this social and emotional child? Because they will be constantly thinking about the child being taken away from them, I don’t think they will even be keen to discuss it.

(GP3) And this is a huge can of worms if we start digging for emotional and [social health] (Dandenong focus group)

## Discussion

We have applied a systematic process to our data analysis with a view to developing an intervention designed to increase preventive healthcare for young children. Despite the fact that our sample populations were sourced from three very diverse socioeconomic backgrounds, we found that within each focus group, participants described a range of experiences from practices both well established with delivering HKCs and others just venturing out with service delivery. All focus groups expressed approval for fiscal-type interventions that maximised participation from population groups likely to be more vulnerable, and all groups discussed the likelihood that HKCs may duplicate services offered by MCHNs. Although small in number, the area where two PNs had established specific HKC-clinics has rates of developmental vulnerability almost twice the state average [6], indicating they may have responded to an increased need in their populations.

Analysis using the TDF afforded a detailed understanding of the barriers and enablers that impact individually, within and external to the general practice environment, and distillation of the findings into the COM-B model has set the stage for developing the components of a complex intervention. Analysis indicates that a number of behaviours could be targeted, including practitioners’ skills and knowledge as well as their beliefs about respective practitioner roles. The opportunities afforded by the mix of practitioners, the roles of support staff, the availability of equipment, and the social milieu created by government policy, suggest additional interventions. These are tabulated and discussed in detail below (Table 6).

At the practitioner level, PNs’ capabilities could benefit from education and skills training that should incorporate interpersonal skills training to overcome their trepidation communicating developmental deficits to parents. The parent sensitivities they described reflect those found in a study of MCHNs, where strong parent-nurse relationships dissipated some of the difficulties experienced discussing weight with parents of young children [33]. From this study, clear demarcation of roles increased PN confidence, and training schemes could utilise respected leaders from both general practice and nursing to model shared roles for delivering different components of the HKC. For example, components of the HKC that incorporate clinical judgement and decision-making may be more appropriate to the role of GPs. Apportioned roles are already a part of Australian general practice where practice nurses assist a supervising GP with aged care health assessments and chronic disease management, using a team-based model of care. This also fits with international processes, outside of the US, where child health surveillance is a divided responsibility between different professionals [34].

Expressions of low confidence with the evidence behind the HKC, ambivalence towards outcomes, and confusion as to why it had been introduced in the first place, explained some of the reluctance of GPs to implement the HKC. Much criticism has been levelled at the low levels of evidence for some of the existing components of the HKC [35] and the inclusion of social and emotional ‘mental’ health assessments [36]. Information and provision of various developmental screening tools would serve to demonstrate the gains to be made when using structured developmental assessments, which have an evidence base for increasing the detection rate and reducing delays [37,38]. This would help to ‘standardise’ social and emotional assessments in particular, an aspect of development practitioners found particularly difficult to assess [39]. Practitioner education needs to be more explicit about the objectives of early intervention, the advances that could be made as well as the limitations of current evidence [40]. Training workshops could be delivered through Medicare Locals, organisations that have previously assisted practices establish ‘chronic disease management’ programmes, and are positively viewed by practitioners as a source of assistance [41].

Findings in relation to the opportunities afforded by the broader social environment indicate key connections between immunisation services and delivery of HKCs. Delivery of a HKC at an earlier age would give more time to intervene early in a child’s development, but primary vaccinations are complete by 18 months, an age too soon for accurate assessment of all aspects of a child’s development. Alternatively, instead of a single health assessment, additional developmental assessments, not tied to vaccination time-points, could be funded to take place in general practice, in keeping with recommendations for a continuous process of child surveillance. Annual assessments, for example, would provide alternative surveillance opportunities where families have prematurely disengaged from MCHN services, although this could risk duplicating services. Alternatively, the co-location of MCHN services within general practice may encourage opportunities for child surveillance in some communities where access is limited [42]. Having a flexible delivery-model for child health prevention is likely to be welcomed by families juggling the demands of child-rearing when both parents work, for example, and may help to overcome the barrier of birth order (subsequent children are less likely than first-borns to receive MCHN services) that we identified in a parallel parent study [17]. Flexible service delivery models were also one factor that contributed to increasing vaccination rates from 53% to more than 90% in the 1990s [43]. In addition, this would send a strong message about the importance of early intervention to both parents and practitioners, with the potential for general practice to significantly contribute towards developmental surveillance. Recommendations designed to overcome other environmental barriers could include the promotion and funding of developmental screening tools suited to the time constraints of primary care services, provision, in paper-format, of health promotion literature, and support for IT tools and equipment that promote the implementation of HKCs.

A major motivator for practitioners was their belief about the consequences of preventive healthcare for young children. Practitioners’ testimonies suggested that the availability, or otherwise, of referral services could enhance or constrain participation in preventive health, and pre-determined referral pathways clearly increased PNs’ confidence to administer HKCs. Dissemination of information about local healthcare services, costs and availability, would reduce the considerable individual effort required by practitioners to establish and maintain up-to-date resource repositories. The experience of a state-wide programme in the US validates linkage of community resources with practices, and was found to be essential for screening to be effective [44].

The fact that the HKC had acted as a ‘catalyst’ to professional development amongst some PNs and GPs suggests that some practitioners were poised to take on an extended role in paediatric healthcare. In addition, several GPs and PNs appeared amenable to practising more preventive healthcare and working alongside childhood educators and MCHNs. Primary care organisations could provide the support for networks of professionals, from different disciplines in child preventive healthcare*,* to develop expertise*,* share information, and build overall capacity. As well as increasing opportunities for collaborative care, this would also strengthen referral pathways. Precedent exists as similar collaborations have been successfully implemented across disciplines in Australian primary mental health care [45] with minimal central funding and ongoing voluntary commitment from a broad array of practitioners.

The barriers identified by this study are similar to those uncovered elsewhere, with notable exceptions. Practitioners were not deterred by inadequate reimbursements for providing HKCs, nor that they lacked the staff to conduct assessments. This may reflect important differences in models of service delivery, as these were barriers expressed by primary care clinicians in the US [16,46], where regular surveillance of children is strongly advocated, but delivered primarily by family physicians and paediatricians, rather than MCHNs. In addition, practitioners did not discuss the use of structured developmental assessments (which are commonly utilised in the US [47]). Whilst practitioners thought that tools specific to primary care practice would be useful, particularly when making assessments about social and emotional development, it was apparent that most practitioners were not aware of the various instruments currently available to them.

### Strengths and limitations

There were several limitations to this study. The TDF was originally designed to be accessible and useful to an interdisciplinary audience to understand behaviours around evidence-based guidelines. The researchers had a combined wealth of experience in general practice, preventive care, and qualitative research methods but did not have access to the skills of a behavioural psychologist. Had we had such access, further insights may have been generated, but in this way we have adhered to the original intent of the TDF. Additionally, the fact that preventive healthcare for children, including HKCs, is based on low levels of evidence could have increased the variation in behaviours, so that some discrepancies may have been missed. The 40 practitioners who took part in the focus groups were likely to be more motivated towards prevention or paediatric health, and less motivated practitioners may have additional deterrents to providing preventive healthcare to young children. This study was, however, purposefully aimed at practitioners working in socio-economically diverse metropolitan suburbs and captured a broad range of behaviours around the provision of HKCs. Focus groups run the risk of introducing bias resulting from an individual’s desire to conform to social acceptability, and their perceptions were not actuality. Further studies, using a mix of quantitative and alternative qualitative methods, could be done to address this, and could obtain the views of practitioners from rural areas and other states where variations in health structures and service delivery may produce different results [48]. The fact that common and significant barriers were detected in this engaged group, however, implies that larger gains are likely to be made where the starting base is low. In addition, the participation rate for the focus groups was adequate, and responses were generated in an iterative process that proceeded across each of the study areas with no new data relevant to the topic of interest generated in the last of the six groups, suggesting that saturation had been obtained. Moreover, feedback, solicited from participants, did not amend the study’s findings.

Despite these limitations, there were considerable strengths in this study. This was the first study to apply the TDF to understand preventive healthcare in young children and therefore adds to the body of work that constitutes knowledge translation research. Moreover, the use of the COM-B model as an additional step in the analysis increased the study’s efficiency and proved that the framework was adequate for purpose. An alternative method would have been to analyse the data within the domains of the TDF as a single step. Previous research has used ‘relevance criteria’ to determine which domains could be targeted by potential interventions [49]. In this study, an unwieldy 11 of 12 domains would have had to be considered, making subjective decisions necessary and potentially causing important evidence to be disregarded.

## Conclusions

Using an evidence-based methodology, we have shown that while the barriers to delivery of preventive healthcare and HKCs are considerable, opportunities do exist for improvement. The TDF has generated an increased awareness of the current situation and has clarified which barriers need to be targeted to improve implementation. As discussed, many interventions could be applied during a single programme, and a pragmatic approach needs to be taken to ensure the ‘recipe for change’ contains the correct ‘measures’ and ‘timing,’ as well as the right ‘ingredients.’ The design and mode of delivery of this complex intervention will combine the findings from previous research with parents [17] and discussion with a group of stakeholders, prior to piloting and further testing in general practice.

### Endnotes

^a^Child health surveillance includes measuring growth and promoting healthy weight, developmental assessments including vision, hearing and social and emotional health, assessments of oral health, injury prevention, and other health promotion activities [50].

^b^The original TDF was reviewed, modified and published in 2012 [31], but because data was collected using the original framework (in 2011), analysis was made according to this framework.

## Competing interests

The authors declare that they have no competing interests.

## Authors’ contributions

DM conceived and co-supervised the study with BB, KA and BB facilitated the focus groups and undertook data analysis, KA wrote the first draft of the manuscript, BB read and critically reviewed the manuscript and all authors read and approved the final manuscript.

## Authors’ information

Karyn E Alexander, PhD candidate, Department of General Practice, School of Primary Health Care, Monash University, Building 3, 270 Ferntree Gully Road, Notting Hill, Melbourne, Victoria 3168, Australia. Bianca Brijnath, NHMRC Early Career Public Health Fellow, Department of General Practice, School of Primary Health Care, Monash University, Building 1, 270 Ferntree Gully Rd, Notting Hill, Melbourne, Victoria 3168, Australia. Danielle Mazza, Head of Department, Department of General Practice, School of Primary Health Care, Monash University, Building 1, 270 Ferntree Gully Rd, Notting Hill, Melbourne, Victoria 3168, Australia.
